# Reported Complications of Bubble Continuous Positive Airway Pressure Systems in Low-Resource Settings: An International Survey

**DOI:** 10.4269/ajtmh.23-0065

**Published:** 2023-05-15

**Authors:** Andrew Geli Wu, Julia R. Klein, Stephen C. John, Tina M. Slusher, Gwenyth A. Fischer, Ann M. Brearley, Ashley R. Bjorklund

**Affiliations:** ^1^ Division of Critical Care Medicine, Department of Anesthesiology, Critical Care and Pain Medicine, Boston Children’s Hospital, Boston, Massachusetts;; ^2^ Department of Medicine, University of Minnesota Medical School, Minneapolis, Minnesota;; ^3^ Department of Pediatrics, University of Minnesota Medical School, Minneapolis, Minnesota;; ^4^ Pediatric Critical Care Medicine, Department of Pediatrics, Hennepin Healthcare, Minneapolis, Minnesota;; ^5^ Division of Pediatric Critical Care Medicine, Department of Pediatrics, University of Minnesota, Minneapolis, Minnesota;; ^6^ Division of Biostatistics, University of Minnesota School of Public Health, Minneapolis, Minnesota

## Abstract

Lower respiratory tract infections (LRTIs) are the leading cause of death in young children globally. Most of the global burden of mortality from LRTIs occurs in low-resource settings (LRSs), where obtaining and maintaining respiratory support devices such as commercial bubble continuous positive airway pressure (bCPAP) can be prohibitive. Low-cost bCPAP devices exist, such as the homemade WHO-style bCPAP design, but the safety of this design has been called into question. Based on our team’s experience with homemade bCPAP, the side effects of the high pressures described in recent studies are not commonly encountered. Therefore, we sought feedback via an international survey about various complications including pneumothorax from practitioners in LRSs who use two forms of homemade bCPAP. In our qualitative survey, we did not find a convincing pattern in the recall of complications between commercial bCPAP and homemade bCPAP with narrow- or wide-bore expiratory limb in neonates or older children.

Lower respiratory tract infections are the leading cause of death in children less than 5 years old worldwide.^1^^,^^2^ This burden is disproportionately high in low-resource settings (LRSs), where respiratory support resources are lacking.^1^^,^^3^ In recent decades, bubble continuous positive airway pressure (bCPAP) has been used successfully, especially in neonates.^4^^,^^5^ With bCPAP, the pressure is set hydrostatically by submerging the expiratory limb to a specified depth (e.g., 5 cm H_2_O). Pressure at the air–water interface equals the density of water multiplied by the gravitational constant and the submerged depth of bubbling. As long as there is laminar flow with minimal resistive pressure losses in the breathing circuit, the pressure delivered to the patient will approximate the pressure at the air–water interface. Neonatal use has been associated with a decreased need for mechanical ventilation and improved outcomes.^6^ Based on this success, many commercial iterations of bCPAP have been developed.^4^^,^^7^^,^^8^ Unfortunately, many commercial bCPAP devices remain relatively expensive for LRSs. As a result, less expensive modified bCPAP versions have been developed, often made of common hospital supplies and constructed onsite, hereafter referred to as “homemade bCPAP.”^9^ A popular version is published in the WHO’s *Oxygen Therapy for Children*.^10^

If the expiratory limb has a narrow internal diameter (e.g., less than 1 cm H_2_O), as is the case with the version described by the WHO, there is concern that resistive pressure losses may lead to a difference between the set pressure (determined by the submerged depth of the bubbling air–water interface) and delivered pressure at the infant’s nares.^11^^,^^12^ For example, if the submerged depth of the bubbling air–water interface is 5 cm H_2_O but there is a 15 cm H_2_O resistive pressure loss in the expiratory circuit due to a narrow internal diameter, the delivered pressure at the nares would be 5 + 15 = 20 cm H_2_O. (Of note, resistive pressure losses are directly proportional to flow as long as the flow is laminar. Also, if there is a nonocclusive seal at the patient’s nares, much of this excess pressure may be leaked to the environment rather than delivered to the patient.) There is concern that the delivery of elevated pressures to the patient could increase the risk of pneumothorax. In our clinical experience in LRSs, we have infrequently encountered cases of pneumothoraces when using homemade bCPAP with a narrow expiratory limb. However, given these reasonable concerns, we conducted a survey among practitioners in LRSs who use bCPAP in neonates/children to estimate the risk of using commercial bCPAP, homemade bCPAP with a narrow expiratory limb, and homemade bCPAP with a wide expiratory limb.

The survey explores the respondents’ recall of complications from bCPAP based on patient age, size of expiratory limb tubing, and type of circuit—homemade or commercial. (The full survey is included in Supplemental Information.) The survey also asked for the role of the survey respondent and the range of monthly admissions for respiratory distress among neonates and older children. Hospital clinician contacts working in low- to middle-income countries (LMICs) identified via contacts of the authors and established University of Minnesota international academic and clinical partnerships were surveyed. Surveys were distributed via e-mail with a templated message explaining the purpose of the voluntary survey.

Responses were collected via Google Forms (Mountain View, CA) and organized in Microsoft^®^ Excel^®^ for Microsoft 365 MSO (16.0.13929.20360) (Redmond, WA) by background information, device type (commercial bCPAP, homemade bCPAP–wide expiratory limb, homemade bCPAP–narrow expiratory limb), and age (neonates, children beyond neonates). Analyses were performed with StataSE 16 and XLMiner Analysis ToolPak (College Station, TX). Descriptive analyses were done. The study was not powered to detect differences, so our results are exploratory in nature.

Of 25 hospitals surveyed, 16 surveys were completed (64% response rate). In neonates, 14 hospitals used homemade bCPAP (7 narrow expiratory limb) and 10 used commercial bCPAP. In children older than 28 days, 10 used homemade bCPAP (6 narrow expiratory limb) and 3 used commercial bCPAP. All 16 hospitals used at least one form of bCPAP. Countries represented included Tanzania, Nepal, Cambodia, Nigeria, Haiti, Somaliland, Togo, Pakistan, Laos, and Kenya. All respondents were physicians.

Specific complications solicited by the survey were nasal irritation, gastric distention, and pneumothorax. The most common complication was nasal irritation and pneumothorax was the least common (Tables 1 and 2).

**Table 1:** Complications of various bCPAP designs among neonates

No. hospitals, cases, and complications	Commercial bCPAP	Homemade bCPAP	
		Narrow limb	Wide limb
Number of hospitals reporting use of a device	10	7	7
Monthly median of respiratory cases among these hospitals	20	20–30	11–20
Remembered frequency of nasal irritation, *n* (%)			
Never	2 (20)	2 (29)	0
Rarely (1/1,000)	0	1 (14)	1 (14)
Sometimes (1/100)	2 (20)	2 (29)	5 (71)
Often (1/10)	6 (60)	2 (29)	1 (14)
Remembered frequency of gastric distension, *n* (%)			
Never	1 (10)	1 (14)	0
Rarely (1/1,000)	0	2 (29)	0
Sometimes (1/100)	5 (50)	2 (29)	5 (71)
Often (1/10)	4 (40)	2 (29)	2 (29)
Remembered frequency of pneumothorax, *n* (%)			
Never	5 (50)	4 (57)	5 (71)
Rarely (1/1,000)	5 (50)	3 (43)	2 (29)
Sometimes (1/100)	0	0	0
Often (1/10)	0	0	0

bCPAP = bubble continuous positive airway pressure. Unless otherwise indicated, numbers displayed in the table are the count of responding hospitals followed by the proportion among all hospitals using the indicated bCPAP design (%).

**Table 2:** Complications of various bCPAP designs among older children (i.e., beyond neonatal age)

No. hospitals, cases, and complications	Commercial bCPAP	Homemade bCPAP	
		Narrow limb	Wide limb
Number of hospitals reporting use of a device	3	6	4
Monthly median of respiratory cases among these hospitals	11–20	> 30	20–30
Occurrence of nasal irritation, *n* (%)			
Never	0	4 (67)	0
Rarely (1/1,000)	0	1 (17)	2 (50)
Sometimes (1/100)	1 (33)	1 (17)	1 (25)
Often (1/10)	2 (67)	0	1 (25)
Occurrence of gastric distension, *n* (%)			
Never	0	3 (50)	0
Rarely (1/1,000)	0	1 (17)	1 (25)
Sometimes (1/100)	3 (100)	2 (33)	3 (75)
Often (1/10)	0	0	0
Occurrence of pneumothorax, *n* (%)			
Never	2 (67)	6 (100)	3 (75)
Rarely (1/1,000)	1 (33)	0	1 (25)
Sometimes (1/100)	0	0	0
Often (1/10)	0	0	0

bCPAP = bubble continuous positive airway pressure. Unless otherwise indicated, numbers displayed in the table are the count of responding hospitals followed by the proportion among all hospitals using the indicated bCPAP design (%).

It appears that commercial bCPAP may incur the highest risk of nasal irritation in the neonatal age group (“often” reported six times) and that homemade bCPAP with a wide expiratory limb incurs the least risk (“often” reported once). In older children, nasal irritation was recalled as being equally as common when using commercial bCPAP compared with using homemade bCPAP with a wide limb. Of note, nasal irritation in older children when using homemade bCPAP with a narrow limb appeared to mostly be recalled as a “never” event.

Gastric distention among neonates using homemade bCPAP with a wide expiratory limb was reported as occurring equally or less commonly compared with the other device types in all frequency categories. In older children, gastric distention seemed to be the least common when using homemade bCPAP with a narrow limb, with it being recalled as a “never” event in three respondents, whereas being recalled as a “sometimes” event three times in the other two device types. Pneumothorax, the most serious potential adverse event in this survey, was mostly reported as “never” or “rarely” in both age groups among all three device types.

Multiple prior studies have demonstrated the efficacy of commercial and homemade bCPAP in neonates and children with lower respiratory tract disease.^4^^,^^6^^–^^9^ However, as noted above, concerns regarding homemade bCPAP have been recently raised, especially for designs with a narrow expiratory limb. Therefore, we conducted this study by surveying multiple hospitals in multiple LMICs to estimate complication rates from various bCPAP designs in neonates/children.

According to the respondents, pneumothorax rarely or never occurred in their experience when using bCPAP in both age groups. Pneumothorax in neonates was reported as a “rare” event in five hospitals when using the homemade versions, which may be consistent with Ettinger et al.’s concerns for high pressures generated in the neonatal airway due to high-resistance dimensions of the expiratory limb.^12^ Lower flow rates (e.g., 2–4 L/per minute instead of 5–10 L/per minute) along with nonocclusive nasal interfaces may be the reason that pneumothoraces are not observed with the homemade WHO-style design as much as may be suspected.

Importantly, these homemade versions of bCPAP may be the only available form of respiratory support outside of low-flow oxygen for many LRSs. Provided that the benefit outweighs known risks, practitioners should strive to use available technologies that optimize patient outcomes while awaiting more resources to become available. Overall, hospitals surveyed in this study reported severe complications as quite rare, which suggests that the WHO-style bCPAP may still provide a favorable benefit–risk ratio, especially when the only alternative is a low-flow nasal cannula.^10^

Limitations of this study include the following. 1) Rates of adverse events were reported in broad categories and are estimates based on subjective recall by providers. 2) We did not ask survey respondents to categorize the medical interventions done as a result of complications to ascertain the severity of the complication. 3) We categorized bCPAP devices into three categories. Variability within a single device “type” exists, making comparisons between a specific commercial bCPAP device and a specific homemade design challenging. 4) The survey did not assign an illness score when bCPAP was applied, making it possible that hospitals with higher acuity patients used more commercial bCPAP. 5) Data were not collected to assess effectiveness (i.e., estimated mortality, length of stay) and therefore our data should not guide decisions regarding bCPAP efficacy. 6) We do not know how many infants and children are represented by each responding hospital.

In a qualitative international survey of hospitals that use commercial and homemade WHO-style bCPAP, respondents recall that the side effects were similar. In the homemade WHO-style bCPAP in children, pneumothorax was largely reported as a “rare” or “never” event based on recall and clinical experience. Next steps should include measuring the actual pressures generated by each of these devices in the neonates and children using them.

## Supplemental Material


Supplemental materials
