# Supplementary material for: Reported Complications of Bubble Continuous Positive Airway Pressure Systems in Low-Resource Settings: An International Survey
Source: Am J Trop Med Hyg. 2023 May 15;109(1):214–6. doi: 10.4269/ajtmh.23-0065 (PMC10324012; doi:10.4269/ajtmh.23-0065)
Supplement: Supplementary file 1 [file tpmd230065.SD1.pdf]

**Use of Bubble CPAP: International Survey**
**Last Updated: 5/18/2022**

**Background Information**

Hospital Location: ________________________________________________________________________
Your role at the hospital (nurse, doctor, administration, etc.): _____________________________________

Approximate number of cases of respiratory distress per month in neonates (age < 29 days) at your institution:
1-5 ☐ 6-10 ☐ 11-20 ☐ 20-30 ☐ >30 ☐
What proportion of these neonates requires respiratory support?
<10% ☐ 10-25% ☐ 25-50%☐ 50-75% ☐ >75% ☐
What are the main causes of respiratory distress?
pneumonia ☐ sepsis ☐ birth asphyxia ☐ neonatal respiratory distress syndrome ☐
bronchiolitis ☐ other☐:______________________

Approximate number of cases of respiratory distress per month in children beyond the neonate:
<10 ☐ 10-20 ☐ 20-30 ☐ >30 ☐ If >30, please estimate: _______
What proportion of these neonates requires respiratory support?
<10% ☐ 10-25% ☐ 25-50%☐ 50-75% ☐ >75% ☐
What are the main causes of respiratory distress?
pneumonia ☐ sepsis ☐ bronchiolitis☐ trauma ☐ asthma☐ upper airway obstruction ☐ other☐:______________________

What is the source of your oxygen (check all that apply)? Bedside concentrator ☐ Oxygen Tank ☐ Other ☐
Do you have access to compressed air? Yes ☐ No ☐

---

**CPAP Questions**

1. Are you using bubble CPAP in neonates (age < 29 days)?

No ☐ – Skip to question 2
Yes ☐ – Continue

What type?
Homemade ☐ (Answer Section 1A)
Commercial ☐ (Answer Section 1B)
Both ☐ (Answer Sections 1A, 1B)

<u>Section 1A</u>
For homemade bubble CPAP, is it made with a nasal cannula? Yes ☐ No ☐

Please describe any oxygen blending used: ____________________________________________
Please describe any humidification used: ______________________________________________

Is the expiratory limb the diameter of a nasal cannula or oxygen tubing? Yes ☐ No ☐ Don't know ☐

If the expiratory limb is NOT the diameter of a nasal cannula or oxygen tubing, please describe what is used or the size of the limb and whether it is larger in diameter than a nasal canula:
________________________________________________________________

What complications have you seen with homemade bubble CPAP with a nasal cannula with narrow diameter expiratory limb (nasal cannula or oxygen tubing) and at what approximate frequency in neonates?

i. Nasal irritation: No ☐ Yes ☐ If yes, rare (1/1000) ☐ sometimes (1/100) ☐ often (1/10) ☐
ii. Gastric distention: No ☐ Yes ☐ If yes, rare (1/1000) ☐ sometimes (1/100) ☐ often (1/10) ☐
iii. Pneumothorax: No ☐ Yes ☐ If yes, rare (1/1000) ☐ sometimes (1/100) ☐ often (1/10) ☐
iv. Other (list): _______________ If yes, rare (1/1000) ☐ sometimes (1/100) ☐ often (1/10) ☐

Is the expiratory limb commercial CPAP tubing or a large diameter tube (at least 10mm)? Yes ☐ No ☐ Don't know ☐

What complications have you seen with homemade bubble CPAP with commercial CPAP tubing or large diameter expiratory limb and at what approximate frequency in neonates?

i. Nasal irritation: No ☐ Yes ☐ If yes, rare (1/1000) ☐ sometimes (1/100) ☐ often (1/10) ☐
ii. Gastric distention: No ☐ Yes ☐ If yes, rare (1/1000) ☐ sometimes (1/100) ☐ often (1/10) ☐
iii. Pneumothorax: No ☐ Yes ☐ If yes, rare (1/1000) ☐ sometimes (1/100) ☒ often (1/10) ☐
iv. Other (list): _______________ If yes, rare (1/1000) ☐ sometimes (1/100) ☐ often (1/10) ☐

<u>Section 1B</u>

Do you use commercial bubble CPAP in the neonate? Yes ☐ No ☐ Don't know ☐
Do you use commercial CPAP (no bubble) in the neonate? Yes ☐ No ☐ Don't know ☐

Please describe any oxygen blending used: _______________________________________________
Please describe any humidification used: _________________________________________________

List brand or brands of commercial bubble CPAP you have for neonates
a.__________________________ b.________________________c.________________________ Don't know ☐

What complications have you seen and approximate frequency with **commercial CPAP in the neonate**

i. Nasal irritation: No ☐ Yes ☐ If yes, rare (1/1000) ☐ sometimes (1/100) ☐ often (1/10) ☐
ii. Gastric distention: No ☐ Yes ☐ If yes, rare (1/1000) ☐ sometimes (1/100) ☐ often (1/10) ☐
iii. Pneumothorax: No ☐ Yes ☐ If yes, rare (1/1000) ☐ sometimes (1/100) ☐ often (1/10) ☐
iv. Other (list): _______________ If yes, rare (1/1000) ☐ sometimes (1/100) ☐ often (1/10) ☐

2. Are you using CPAP in children beyond the neonate (age > 28 days up to 5 years)?
No ☐ – This survey is complete
Yes ☐ – Continue

What is the upper weight (kg) or age limit (yr/mo) you routinely use it on? _____________

What type?
Homemade ☐ (Answer Section 2A)
Commercial ☐ (Answer Section 2B)
Both ☐ (Answer Sections 2A, 2B)

Section 2A

For homemade bubble CPAP, is it made with a nasal cannula? Yes ☐ No ☐

Please describe any oxygen blending used: ______________________________________________
Please describe any humidification used: ________________________________________________

Is the expiratory limb the diameter of a nasal cannula or oxygen tubing? Yes ☐ No ☐ Don't know ☐

If the expiratory limb is NOT the diameter of a nasal cannula or oxygen tubing, please describe what is used or the size of the limb and whether it is larger in diameter than a nasal canula:
_____________________________________________________________________

What complications have you seen with homemade bubble CPAP with a nasal cannula with narrow diameter expiratory limb (nasal cannula or oxygen tubing) and at what approximate frequency in children beyond the neonate?

i. Nasal irritation: No ☐ Yes ☐ If yes, rare (1/1000) ☐ sometimes (1/100) ☐ often (1/10) ☐
ii. Gastric distention: No ☐ Yes ☐ If yes, rare (1/1000) ☐ sometimes (1/100) ☐ often (1/10) ☐
iii. Pneumothorax: No ☐ Yes ☐ If yes, rare (1/1000) ☐ sometimes (1/100) ☐ often (1/10) ☐
iv. Other (list): ________________ If yes, rare (1/1000) ☐ sometimes (1/100) ☐ often (1/10) ☐

Is the expiratory limb commercial CPAP tubing or a large diameter tube (at least 10mm)? Yes ☐ No ☐ Don't know ☐

What complications have you seen with homemade bubble CPAP with commercial CPAP tubing or large diameter expiratory limb and at what approximate frequency in children beyond the neonate?

i. Nasal irritation: No ☐ Yes ☐ If yes, rare (1/1000) ☐ sometimes (1/100) ☐ often (1/10) ☐
ii. Gastric distention: No ☐ Yes ☐ If yes, rare (1/1000) ☐ sometimes (1/100) ☐ often (1/10) ☐
iii. Pneumothorax: No ☐ Yes ☐ If yes, rare (1/1000) ☐ sometimes (1/100) ☐ often (1/10) ☐
iv. Other (list): ________________ If yes, rare (1/1000) ☐ sometimes (1/100) ☐ often (1/10) ☐
v.

Section 2B

Do you use commercial bubble CPAP in children beyond the neonate? Yes ☐ No ☐ Don't know ☐
Do you use commercial CPAP (no bubble) in children beyond the neonate? Yes ☐ No ☐ Don't know ☐

Please describe any oxygen blending used: ______________________________________________
Please describe any humidification used: ________________________________________________

List brand or brands of commercial bubble CPAP you have for children beyond the neonate
a.___________________________ b.________________________c.________________________ Don't know ☐

What complications have you seen and approximate frequency with commercial CPAP in children beyond the neonate

i. Nasal irritation: No ☐ Yes ☐ If yes, rare (1/1000) ☐ sometimes (1/100) ☐ often (1/10) ☐
ii. Gastric distention: No ☐ Yes ☐ If yes, rare (1/1000) ☐ sometimes (1/100) ☐ often (1/10) ☐
iii. Pneumothorax: No ☐ Yes ☐ If yes, rare (1/1000) ☐ sometimes (1/100) ☐ often (1/10) ☐
iv. Other (list): ________________ If yes, rare (1/1000) ☐ sometimes (1/100) ☐ often (1/10) ☐

---

**Photos**

Please include at least 1 photo that captures the following four elements of your CPAP device(s). Include as many photos as needed to capture all four elements. Do not take a picture of it on a patient.

- ☐ Oxygen blending device
- ☐ Humidifier
- ☐ Nasal prongs
- ☐ Expiratory limb

Do we have your permission to use these photo(s) in a publication? If so, please give a name to acknowledge.

No ☐ Yes ☐

Acknowledgement: ________________________________
